# Rodents as Sentinels for *Toxoplasma gondii* in Rural Ecosystems in Slovakia—Seroprevalence Study

**DOI:** 10.3390/pathogens12060826

**Published:** 2023-06-12

**Authors:** Daniela Antolová, Michal Stanko, Júlia Jarošová, Dana Miklisová

**Affiliations:** Institute of Parasitology SAS, Hlinkova 3, 040 01 Košice, Slovakia; stankom@saske.sk (M.S.); jarosova@saske.sk (J.J.); miklis@saske.sk (D.M.)

**Keywords:** *Toxoplasma gondii*, rodents, seropositivity, age, sex, sexual activity

## Abstract

*Toxoplasma gondii* is a ubiquitous intracellular parasite with felids as definitive hosts and a broad range of intermediate hosts. Rodents are considered suitable sentinels for prevalence studies of many infections, including toxoplasmosis. This study aimed to estimate the seroprevalence of *T. gondii* in rodents from different localities of Slovakia and investigate the correlation between the seropositivity and the species, age, sex, and sexual activity of animals. Altogether, 1009 wild rodents belonging to 9 species were trapped in 2015 and 2019, and antibodies to *T. gondii* were detected in 6.7% of the animals. Seropositivity was detected in seven species, ranging from 0.0% in *Micromys minutus* and *Apodemus sylvaticus* to 7.7% in *A. flavicollis*. The females reached significantly higher seropositivity (9.7%) than the males (3.8%), and the adults were positive significantly more often (9.2%) than the subadults (4.9%). The seropositivity differed also among localities, with significantly higher positivity detected in suburban and touristic areas (12.2%) than in localities with a lower level of human activities (5.5%). This study showed that the occurrence of *T. gondii* varies significantly in rodent species and habitats with various environmental conditions and different levels of anthropic use. Several biological and ecological factors, e.g., soil contamination, soil conditions, the susceptibility of rodent species etc., may influence this variability.

## 1. Introduction

Free-living small mammals constitute the most abundant and diversified group of all living mammals and act as reservoirs of different pathogen species. Rodents, as the major prey of felids, are considered to play a key role in the maintenance of the *Toxoplasma gondii* life cycle [1,2].

*T. gondii* is an obligate intracellular protozoan parasite with a worldwide distribution. In its definitive hosts, i.e., members of the Felidae family, it has a sexual cycle, while in intermediate hosts it proliferates in an asexual cycle. All warm-blooded animals can act as intermediate hosts and may become infected after the ingestion of any of the three infective stages: tachyzoites, bradyzoites, or sporozoites.

*T. gondii* infection is common in humans, who may develop an infection by the accidental ingestion of sporulated oocysts from the environment or by the consumption of raw or undercooked meat and meat products containing tissue cysts. Its prevalence strongly differs with the geographic and socioeconomic conditions, hygiene level, and eating/cooking habits of the studied population, globally ranging from less than 10% to more than 60% [3]. The disease in healthy adults is often without clinical signs and does not cause serious problems. However, if first contracted during pregnancy, *T. gondii* may cause abortion, neonatal death, or fetal abnormalities, and in immunocompromised patients (AIDS patients, patients receiving immunosuppressive therapy, etc.), severe disease or even fatal toxoplasmosis may occur [4,5].

The course of infection in food-producing animals depends on the animal species. While clinical toxoplasmosis in pigs and cattle is rare, sheep and goats are highly susceptible, and *T. gondii* infection is considered a major cause of reproductive losses in small ruminants worldwide [6]. Moreover, infected animals may represent a risk for the transmission of toxoplasmosis to humans, either directly or through farming [7].

Rodents, as intermediate hosts, play an important role in the circulation of *T. gondii*. Once a rodent is infected, a persistent chronic infection, characterized by the presence of tissue bradyzoites, develops. Therefore, they can be considered suitable sentinels for the prevalence and distribution studies of toxoplasmosis [8,9].

The present study aimed to estimate the seropositivity to *T. gondii* in rodents from different localities of Slovakia and investigate the correlation between the seroprevalence of infection and the species of animals, their age, sex, and sexual activity.

## 2. Materials and Methods

### 2.1. Study Sites and Sampling of Rodents

Free-living rodents were trapped in 2015 and 2019 in seven suburban and rural localities of eastern and central Slovakia. The basic characteristics of the selected localities are presented in Table 1. Agricultural or forestry areas located outside towns with a low human population density were characterized as rural; forests and parks in the peripheral parts of Košice town were considered localities of a suburban type. The proportionality of the trapping efforts in all the localities in terms of the season was tried to be ensured depending on the personnel and technical possibilities.

Standard live and snap traps set in lines, with wicks soaked in an oil and nut mixture used as baits, were used to catch animals. The traps were spaced approximately 5 m apart; each trap line was usually exposed for two nights and was checked regularly in the morning. The captured animals were humanely euthanized in accordance with licenses from the Ministry of Environment of the Slovak Republic, No. 6743/2012-2.1 and No. 4874/2018-2.2.

The rodents were teriologically identified to species level according to their morphological features (size of body, tail, ears, legs, etc.). The sex (male, female), sexual activity (active and inactive), and age classes (subadult, adult) were determined using the identification keys described by Pelikán [10,11]. Concerning the age categories, the animals were classified as subadult (or immature) or adult (sexually mature). The sexual conditions (sexually active vs. inactive) of individuals were ascertained during the dissection after the trapping and anesthesia. In the females, the condition of their ovaries and uteri and the presence of a vaginal plug, embryos, and placental scars were observed. In the males, the size of the testes and the length of the *glandulae vesiculares* were analyzed [10,11].

During necropsy, the hearts with their blood clots were cut open; the sera were prepared as described previously by Antolová et al. [12] and stored at −80 °C until further analysis.

### 2.2. Detection of Antibodies to Toxoplasma gondii

An enzyme-linked immunosorbent assay (ELISA) was used to detect the antibodies to *T. gondii* in the sera. The sensitivity of the ELISA method with goat anti-mouse polyvalent antibodies was validated by several studies on the sera of various species of small mammals [12,13,14,15,16].

A commercially available *Toxoplasma gondii* antigen (CD Creative Diagnostics, New York, NY, USA) prepared from an RH strain [17,18] was used for antibody detection. Microtiter ELISA plates (Nunc; Thermo Fisher Scientific, Roskilde, Denmark) were coated with 100 μL/well antigen diluted in carbonate buffer (pH 9.6) and left to stand overnight at 4 °C. The optimal antigen and conjugate concentrations were determined by previous titrations with the sera of experimentally infected laboratory mice, and the final dilution of the antigen was 3.4 μg protein/mL of dilution buffer. The plates were washed three times with distilled water/0.05% Tween-20. Then, 100 μL of the sera, a negative control serum from uninfected laboratory mice, and positive serum from *A. agrarius* (detected during a previous study) diluted in 5% non-fat dry milk in a phosphate buffer (PBS; pH 7.2) were filled into the wells, and the plates were incubated for 1 h at 37 °C and washed afterwards, as described previously. Goat anti-mouse antibodies (Anti-Mouse Polyvalent Immunoglobulins (G, A, M)—peroxidase antibody produced in goat; Sigma-Aldrich, St. Louis, MO, USA) diluted in PBS were used as the conjugate. After incubation (1 h/37 °C) and a washing step, the reactions were visualized by adding 100 μL of substrate (o-phenylenediamine/methanol with 0.05% H_2_O_2_). The reaction was stopped after 20 min using 50 µL of 4N H_2_SO_4_, and the optical densities (OD) were read at 490 nm.

Since no positive and negative control sera from *T. gondii*-infected wild rodents were available, the cut-off value was determined according to Naguleswaran et al. [19]. The first cut-off value was determined by the mean OD of all the sera plus 3 standard deviations (SD). The sera with an OD above this value were excluded, and the remaining sera were used for the calculation of the mean absorption of the negative samples (Mneg) and SDneg. The sera with OD values above Mneg + 4 SDneg were considered to be positive.

### 2.3. Statistical Analyses

The seroprevalence values are given with 95% confidence intervals (95% CI). Fisher’s exact test was used to test the differences between couples of prevalences for the age, sex and sexual activity of the animals. Fisher’s exact test with a value determined by simulation, using 20,000 Monte Carlo replications, was used to test the differences among the host species. This test was also used to analyze the differences in the seropositivity of animals from different localities. The percentage of positivity and 95% CI were not calculated for “groups” consisting of less than 10 animals. The statistical analyses were performed by Quantitative Parasitology on the Internet [20].

## 3. Results

In total, 1009 wild rodents were trapped in 2015 and 2019, and antibodies to *T. gondii* were detected in 68 animals reaching the overall seroprevalence of 6.7%. The animals belonged to nine species. Six species were classified as belonging to the Muridae family, namely *Apodemus agrarius*—black-striped field mouse (Pallas, 1711), *A. flavicollis*—yellow-necked mouse (Melchior, 1834), *A. sylvaticus*—wood mouse (Linnaeus, 1758), *A. microps*—pygmy field mouse (Kratochvíl and Rosický, 1952), *Micromys minutus*—harvest mouse (Pallas, 1911), and *Mus musculus*—house mouse (Linaeus, 1758). Three species belonged to the Cricetidae family: *Myodes glareolus*—bank vole (Schreber, 1780), *Microtus arvalis*—common vole (Pallas, 1778), and *Microtus subterraneus*—European pine vole (de Sélys-Longchaps, 1836).

Antibodies to *T. gondii* were detected in seven species, with the seropositivity rate ranging from 0.0% in *A. sylvaticus* and *M. minutus* to 7.7% in *A. flavicollis*, but the differences were not of statistical significance (*p* = 0.56). Representatives of the Muridae family were positive slightly more often (7.0%) than members of the Cricetidae family (6.0%), but the observed difference was also not significant (*p* = 0.31) (Table 2).

When analyzing the seropositivity according to the age, sex, and sexual activity of the animals, significant differences were confirmed between the adult and subadult animals (*p* = 0.009), with the adults being positive more often than the subadults (Table 3). Similarly, gender influenced the seropositivity of the rodents significantly, as the female hosts were positive significantly more often than the male ones (*p* = 0.0002) (Table 4). On the other hand, the current state of sexual activity did not significantly affect the seroprevalence of toxoplasmosis; the sexually active animals were positive only slightly more often (8.7%) than the inactive ones (5.5%) (*p* = 0.09).

The difference between the overall seropositivity of the rodents in 2015 and 2019 was statistically significant (*p* = 0.01). The animals trapped in 2019 were positive more frequently (8.8%) than those trapped in 2015 (4.9%). Although the trend of higher positivity in 2019 was recorded also in individual species, a significant difference (*p* = 0.01) was recorded only between the prevalence of antibodies in *A. agrarius* (Table 5).

Significant differences were also found between the seropositivity of rodents trapped in different localities (*p* = 0.02), with the highest positivity detected in Pieniny (16.4%), a sub-mountainous touristic locality localized in the alluvium of the Dunajec river, and the lowest (3.4%) in Kechnec, a rural locality characterized mainly as agrocenosis.

When comparing localities based on the level of anthropic use, the positivity detected in suburban and touristic areas (Košice-suburban zone, Košice-alluvium, and Pieniny) (12.2%) was significantly (*p* = 0.001) higher than the positivity (5.5%) detected in localities with a lower level of human activities (Šebastovce, Kechnec, Rozhanovce, and Poiplie) (Table 6).

## 4. Discussion

Rodents are considered suitable sentinels for prevalence studies of many infections since they are widespread in most ecological systems, have a small home range, and are easy to trap and monitor [21]. Due to the life-long survival of *T. gondii* bradyzoites in tissues, infected intermediate hosts play an important role in the circulation of this parasite in the domestic and sylvatic cycle. Rodents, therefore, present an important source of infection for their predators, including cats, felids, and other carnivores, as well as wild boars and different scavenging species. Although they do not pose a particular threat to human communities, the predation of rodents by cats may cause subsequent contamination of households and other human-related environments [22,23].

In this study, we analyzed the seroprevalence of *T. gondii* in sylvatic rodent populations derived from different localities in Slovakia. Seropositivity to *T. gondii* was recorded in all seven monitored localities, and in seven out of nine rodent species trapped during the study, resulting in an overall seroprevalence of 6.7%. This suggests the ubiquitous occurrence of *T. gondii* in different ecosystems of Slovakia.

The highest seropositivity was detected in *Apodemus flavicollis* (7.7%), while no antibodies were present in *A. sylvaticus* and *Micromys minutus*. However, only 17 individuals of *M. minutus* and 1 of *A. sylvaticus* were examined, which may contribute to the negative result of the survey for these species. In all the other species, the seropositivity ranged from 2.2% to 7.6%, which is in line with the prevalence recorded by some other authors. According to a meta-analysis carried out by Galeh et al. [24], the overall seroprevalence of antibodies to *T. gondii* detected in rodents was 6.0%. In Europe, the positivity rates differ with the country and method used for the detection of antibodies, ranging from 0.0% in Austria [25] to 28.9% in Italy [26]. In Geneva, Switzerland, the overall seroprevalence of *T. gondii* in four monitored rodent species was 5.0% [14], in north-eastern France it ranged from 0.0% to 12.9% [2], and in Poland, Grzybek et al. [16] reported 5.5% seropositivity in four vole species.

The biological characteristics, for example different susceptibility of rodent species and the vertical transmission of *T. gondii* may explain the differences in its seroprevalence among species. Variations in the susceptibility to *T. gondii* were observed in commensal species, e.g., rats being more resistant to infection than mice [27]. Although mostly not tested experimentally, such differences are highly probable in wild species as well. The congenital transmission of *T. gondii* that has been documented in several rodent species, e.g. in *M. domesticus*, *M. musculus*, *A. sylvaticus*, and *Peromyscus maniculatus* [28,29,30], can be also the reason for inter-species variations or, in some cases, higher positivity in young animals compared to adults.

In the present study, the overall seropositivity to *T. gondii*, as well as the seropositivity of individual species except for *M. glareolus*, was higher in the adults than in the subadults. This result suggests that the risk of acquiring toxoplasmosis increases, with a higher probability of exposure to oocysts in the environment, during the animal’s lifetime. A significant relationship between the greater age of rodents and the prevalence of infection has been detected by several authors. In the study of Reperant et al. [14], seropositivity reached 6.4% in adult rodents and 2.1% in subadults. Dabritz et al. [9] reported that the odds ratio for being seropositive was 3.10 for adults compared to juveniles, and older bank voles were seropositive more often (6.1%) than young adults (3.5%) in the study from Poland [16].

In our research, female rodents were infected significantly more often (9.7%) than male rodents (3.8%), and this phenomenon was observed in all seven seropositive species. Similarly, female bank voles were infected more frequently (5.5%) than males (1.6%) in the study of Grzybek et al. [16]. We suppose that a higher food intake during gravidity and lactation could increase the possibility of becoming infected. On the other hand, the prevalence of toxoplasmosis does not seem to considerably depend on the sexual activity of animals. Although sexually active animals were positive more often (8.7%) than inactive ones (5.5%), the observed difference was not statistically significant. Thus, we can conclude that the current state of sexual activity affects the positivity of animals less than their gender and age.

The infection rates varied significantly also among the different trapping localities. The highest seropositivity was detected in Pieniny (16.4%), a sub-mountainous touristic locality localized in the alluvium of the Dunajec River, followed by the Košice alluvium (10.9%), and the Košice suburban zone (9.8%). Two of these locations (Pieniny and Košice alluvium) are characterized as touristic, and the third is a suburban zone. Therefore, they are more frequently and intensively used by humans than the other four areas. The differences in the positivity rate in the locations with a higher (12.2%) and lower (5.5%) level of anthropic load were statistically significant. The finding of a higher positivity to *T. gondii* in rodents sampled near human residences and in touristic localities agreed with the results of a survey conducted in British Columbia, Canada in which 6.3% deer mice (*Peromyscus maniculatus*) from non-residential areas were positive compared to 15.5% positive mice from localities near human residences [31]. Similarly, in the United Kingdom, 59% of mice living in urban environments were infected with *T. gondii* compared to 46% of mice captured in rural habitats [9].

Several biological, ecological, and spatial aspects may affect these variances. The presence of cats or felids is a factor that can directly influence the occurrence of *T. gondii*, as a higher density of cats increases the potential for contamination of the soil with *T. gondii* oocysts. However, some authors did not find a correlation between cat density and the seroprevalence of toxoplasmosis in rodents. Thomasson et al. [32] found unexpected high seropositivity (40.78%) to *T. gondii* in a population of *A. sylvaticus* in an area where the density of cats was very low, and Dabritz et al. [9] observed that rodents caught near residential housing with the presence of free-ranging cats were almost 40% less likely to be seropositive for *T. gondii* compared to those trapped more than 200 m away from residences. Thus, the question regarding the differences in the prevalence of infection in rodents in areas with high and low cat densities remains unanswered [32]. In the present study, we do not have information on the density of cats in the monitored localities; therefore, we cannot discuss the observed differences from this point of view. An additional factor that could influence the differences among the trapping areas is the soil condition (humidity, temperature), which varies significantly and may strongly affect oocyst survival. For example, a higher soil humidity along rivers and brooks could contribute to the detected higher seroprevalence of antibodies in alluvial habitats (Košice alluvium and Poiplie). According to some authors, oocysts are able to survive in moist soil for up to one year [33], especially if they are protected from ultraviolet light [34]. Higher seropositivity in animals exposed to humid and water environments was also described, e.g., by Dabritz et al. and Poulsen et al. [9,35].

Variations in environmental, climate, and ecological conditions are likely responsible for the differences in the seropositivity of the animals trapped in 2015 and 2019. Although the sampling sites were the same, the situation changed, even within one year, depending on the temperature, humidity, or amount of precipitation. The use of agricultural areas situated near the trapping sites is another important factor influencing the situation. Changes in the cultivation of agricultural plants, which are more or less attractive for rodents, can strongly influence the population density of rodents that can give birth to 2–3 litters of young per year and, consequently, the spread of pathogens, including *T. gondii*.

However, there are also some limitations of this research that may have biased the outcomes of the study, resulting in an overestimation or underestimation of the seropositivity. Antibody production can be influenced by various factors, such as the susceptibility of the species, the parasite genotype, the infection persistence, and the host’s age [2,24], as well as the health and nutritional status of the host. The duration of the immune response may vary in some species, and antibodies can become unrecognizable after a period of time [24]. However, this is a general issue of a serological (e.g., ELISA) approach since it is an indirect diagnostic method that can detect the reaction (stronger or weaker) of an organism to an antigen stimulus. Although the estimation of the seroprevalence of infection may not reflect the actual number of infected individuals, the study indicates the ubiquitous occurrence and long-term circulation of *T. gondii* in different ecosystems of Slovakia.

The presented data strongly suggest that the species and geographic variability in *T. gondii* incidence are rather common in rodents. Besides the density of felids, the soil conditions, and the congenital transmission of *T. gondii*, the different susceptibilities of rodent species may explain the variability in the prevalence found between the species and the geographical areas.

## 5. Conclusions

The present study showed that the occurrence of *T. gondii* in different rodent species and localities with different environmental conditions can differ significantly. Several biological and ecological factors, e.g., the level of soil contamination, the soil conditions, the different susceptibilities of rodent species, etc., may influence this variability. Rodents present an important reservoir of infection for domestic and wild felids, which can lead to spillover between the domestic and rural cycle, increasing the risk of infection for both humans and pets. Monitoring of the seroprevalence of *T. gondii* represents a suitable indicator of environmental contamination of the infective stages of this parasite.

## Figures and Tables

**Table 1:** Characteristics of trapping sites of rodents.

Locality	Geographical Coordinates	Altitude (m a.s.l.)	Locality Type	Description
Šebastovce	48°39′18″ N 21°16′13″ E	209	R	Poplar windbreak with agrocenoses on both sides
Kechnec	48°32′57″ N 21°15′52″ E	172	R	Agrocenosis and the edge of poplar windbreak
Rozhanovce	48°45′00″ N 21°21’00″ E	215	R	Mixed forest, hunting ground with pheasants and fallow deer breeding
Košice-suburban	48°44′49″ N 21°14′89″ E	208	S	Hornbeam-oak park with shrubs
Košice-alluvium	48°45’67″ N 21°88’17″ E	238	ST	Suburban recreational locality with mixed forests and shrubs
Pieniny	49°24’11″ N 20°26’13″ E	450	RT	Sub-mountain alluvium of Dunajec River with shrubs
Poiplie	48°05’10″ N 19°27’45″ E	600	R	Lowland alluvium of Ipeľ River with shrubs; situated near fields

a.s.l.—above sea level; R—rural; RT—rural touristic; S—suburban; ST—suburban touristic.

**Table 2:** Seroprevalence of *Toxoplasma gondii* in examined rodents.

Species	N/n	Prevalence (%) (95% CI)	Family (%) (95% CI)
*Apodemus agrarius*	33/451	7.3 (5.1–10.1)	Muridae 7.0 (5.3–9.1)
*Apodemus flavicollis*	17/222	7.7 (4.5–12.0)	
*Apodemus sylvaticus*	0/1	nc	
*Apodemus microps*	1/45	2.2 (0.0–11.8)	
*Micromys minutus*	0/17	0.0 (0.0–19.5)	
*Mus musculus*	1/5	Nc	
*Myodes glareolus*	8/173	4.6 (2.0–8.9)	Cricetidae 6.0 (3.5–9.5)
*Microtus arvalis*	7/92	7.6 (3.1–15.1)	
*Microtus subterraneus*	1/3	nc	
Total	68/1009	6.7 (5.3–8.5)	-

N—number of positive; n—number of tested; nc—not calculated.

**Table 3:** Seroprevalence of *Toxoplasma gondii* in different rodent species from Slovakia according to the age of animals.

Species	Adult		Subadult	
	N/n	Prevalence (%) (95% CI)	N/n	Prevalence (%) (95% CI)
*Apodemus agrarius*	18/156	11.5 (7.0–17.6)	14/288	4.8 (2.7–8.0)
*Apodemus flavicollis*	11/132	8.3 (4.2–14.4)	5/84	5.9 (2.0–13.4)
*Apodemus sylvaticus*	0/1	nc	0/0	nc
*Apodemus microps*	1/24	4.2 (0.1–21.1)	0/21	0.0 (0.0–16.1)
*Micromys minutus*	0/2	nc	0/15	0.0 (0.0–21.8)
*Mus musculus*	1/2	nc	0/1	nc
*Myodes glareolus*	2/61	3.2 (0.4–11.4)	6/99	6.1 (2.3–12.7)
*Microtus arvalis*	5/37	13.5 (4.5–28.8)	2/55	3.6 (0.4–12.5)
*Microtus subterraneus*	0/0	nc	1/3	nc
Total	38/415	9.2 (6.6–12.4)	28/566	4.9 (3.3–7.1)

N—number of positive; n—number of tested; nc—not calculated.

**Table 4:** Seroprevalence of *Toxoplasma gondii* in rodents according to the sex and sexual activity of animals.

Species	Male		Female		Sexually Active		Sexually Inactive	
	N/n	% (95% CI)	N/n	% (95% CI)	N/n	% (95% CI)	N/n	% (95% CI)
*Apodemus agrarius*	10/231	4.3 (2.1–7.8)	22/213	10.3 (6.6–15.2)	10/97	10.3 (5.1–18.1)	20/332	6.0 (3.9–9.2)
*Apodemus flavicollis*	5/121	4.1 (1.4–9.4)	11/96	11.5 (5.9–19.6)	3/53	5.7 (1.2–15.7)	10/148	6.8 (3.3–12.1)
*Apodemus sylvaticus*	0/1	nc	0/0	nc	0/1	nc	0/0	nc
*Apodemus microps*	0/24	0.0 (0.0–14.3)	1/21	4.8 (0.1–23.8)	1/12	8.3 (0.2–38.5)	0/28	0.0 (0.0–10.2)
*Micromys minutus*	0/9	nc	0/8	nc	0/0	nc	0/16	
*Mus musculus*	1/4	nc	0/1	nc	1/2	nc	0/1	nc
*Myodes glareolus*	2/86	2.3 (0.3–8.2)	6/87	6.9 (2.6–14.4)	1/40	2.5 (0.0–13.2)	6/116	5.2 (1.9–10.9)
*Microtus arvalis*	1/43	2.3 (0.0–12.3)	6/49	12.2 (4.6–24.8)	3/14	21.4 (4.7–50.8)	2/61	3.3 (0.4–11.2)
*Microtus subterraneus*	1/2	nc	0/1	nc	0/0	nc	1/3	nc
Total	20/521	3.8 (2.4–5.9)	46/476	9.7 (7.2–12.9)	19/219	8.7 (5.3–13.2)	39/705	5.5 (4.0–7.5)

N—number of positive; n—number of tested; %—prevalence; nc—not calculated.

**Table 5:** Seroprevalence of *Toxoplasma gondii* in different rodent species in 2015 and 2019.

Species	Year 2015		Year 2019		*p*
	N/n	% (95% CI)	N/n	% (95% CI)	
*Apodemus agrarius*	10/231	4.3 (2.1–7.8)	23/220	10.5 (6.7–15.3)	0.01
*Apodemus flavicollis*	8/128	6.3 (2.7–11.9)	9/94	9.6 (4.5–17.4)	0.35
*Apodemus sylvaticus*	0/0	nc	0/1	nc	nc
*Apodemus microps*	0/27	0.0 (0.0–10.5)	1/18	5.6 (0.1–27.3)	nc
*Micromys minutus*	0/9	nc	0/8	0.0 (0.0–25.9)	nc
*Mus musculus*	1/1	nc	0/4	nc	nc
*Myodes glareolus*	2/80	2.5 (0.3–8.7)	6/93	6.5 (2.4–13.5)	0.21
*Microtus arvalis*	4/52	7.7 (2.1–18.5)	3/40	7.5 (1.6–20.4)	0.97
*Microtus subterraneus*	1/2	nc	0/1	nc	nc
Total	26/530	4.9 (3.2–7.1)	42/479	8.8 (6.4–11.7)	0.01

N—number of positive; n—number of tested; %—prevalence; nc—not calculated.

**Table 6:** Seroprevalence of *Toxoplasma gondii* in rodents from different localities of Slovakia.

Locality	Year 2015		Year 2019		Total	
	N/n	Prevalence (%) (95% CI)	N/n	Prevalence (%) (95% CI)	N/n	Prevalence (%) (95% CI)
Šebastovce	4/108	3.7 (1.0–9.2)	3/40	7.5 (1.2–20.4)	7/148	4.7 (1.9–9.5)
Kechnec	1/69	1.4 (0.0–7.8)	3/47	6.4 (1.3–17.5)	4/116	3.4 (1.0–8.6)
Rozhanovce	7/231	3.0 (1.2–6.1)	22/234	9.4 (6.0–13.9)	29/477	6.1 (4.1–8.6)
Košice-suburban zone	3/36	8.3 (1.8–22.5)	5/46	10.9 (3.6–23.6)	8/82	9.8 (4.3–18.3)
Košice-alluvium	0/0	nc	5/46	10.9 (3.6–23.6)	5/46	10.9 (3.6–23.6)
Pieniny	10/61	16.4 (8.2–28.1)	0/0	nc	10/61	16.4 (8.2–28.1)
Poiplie	1/25	4.0 (0.1–20.4)	4/54	7.4 (2.1–17.9)	5/79	6.3 (2.1–14.2)
Total	26/530	4.9 (3.2–7.1)	42/467	9.0 (6.6–12.0)	68/1009	6.7 (5.3–8.5)

N—number of positive; n—number of tested; nc—not calculated.

## Data Availability

Data sharing not applicable.
